# Supplementary material for: A New Formulation for Strigolactone Suicidal Germination Agents, towards Successful Striga Management
Source: Plants (Basel). 2022 Mar 18;11(6):808. doi: 10.3390/plants11060808 (PMC8955415; doi:10.3390/plants11060808)
Supplement: Supplementary file 1 [file plants-11-00808-s001.zip › Supplementary Figures.pdf]

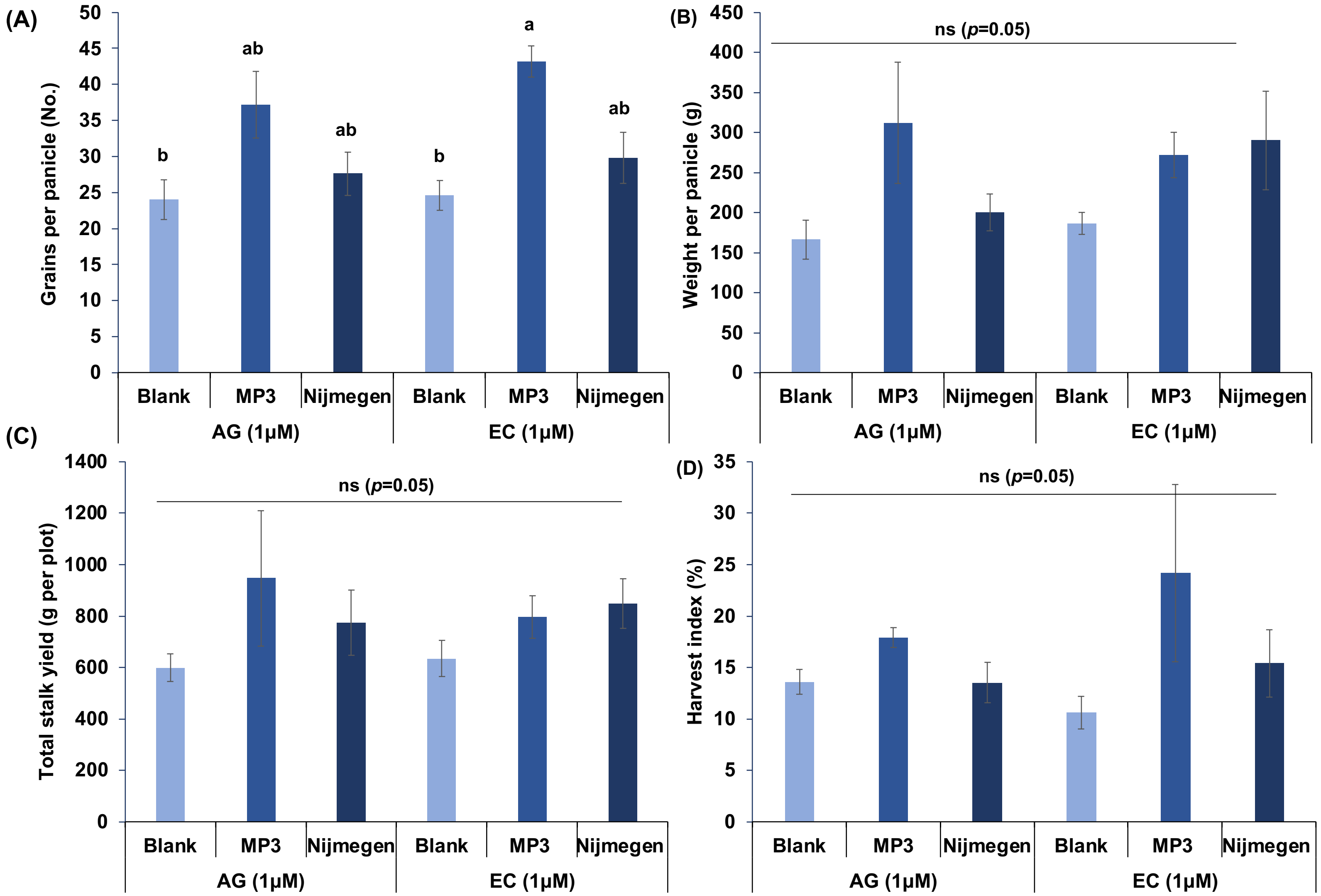

Supplementary Figure S1. Effect of various formulations of SL analogs on number of grains per panicle, panicle dry biomass, total stalk yield and harvest index in Pearl millet field at INERA, Burkina Faso. (**A**) Average grains per panicle (**B**) Average weight of dry panicle (**C**) Average stalk yield and (**D**) Average of harvest index from *Striga* infested Pearl millet field in response to formulated MP3 and Nijmegen application. Both SL analogs of EC and AG formulations (at 1.0 µM) were applied in *Striga* infested Pearl millet. Yield and yield component were measured after 110 days of pearl millet planting. Data are means ± SE (n=5). For each SL analog, treatments with various letters differ significantly ($p < 0.05$). Values in parenthesis are showing the percentage increase (+) or decrease (-) over blank treatment. *ns*: non-significant.

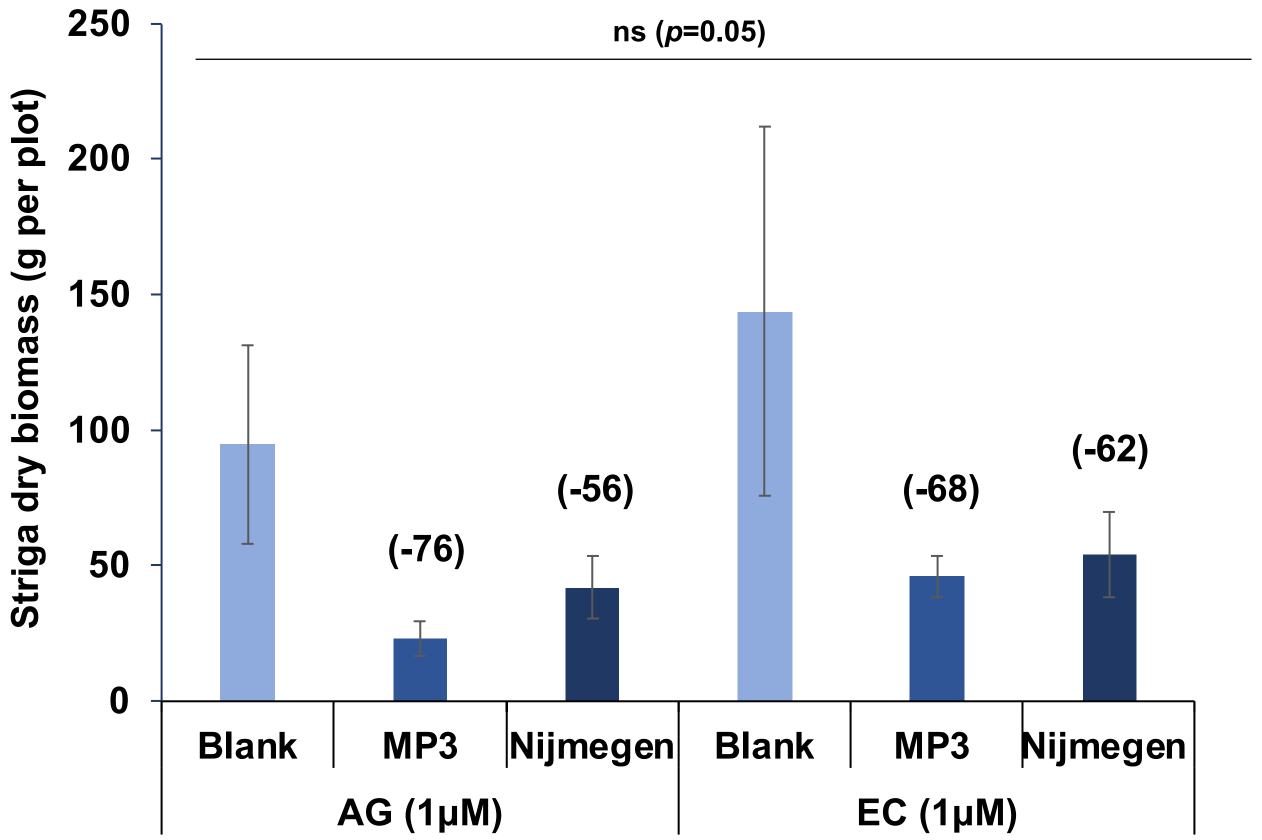

Supplementary Figure S2. Effect of various formulations of SL analogs on *Striga* dry biomass at Burkina Faso. Average *Striga* dry biomass emerged in Sorghum field in response to formulated MP3 and Nijmegen application. Both SL analogs of EC and AG formulations (at 1.0 μM) were applied in *Striga* infested Sorghum fields. *Striga* seedlings were collected from Sorghum infested field and *Striga* dry biomass was measured at final harvest. Values of each bar showed average of *Striga* dry biomass emerged per plot. Data are means ± SE (n=5). For each SL analog, treatments with various letters differ significantly (p < 0.05). Values in parenthesis are showing the percentage increase (+) or decrease (-) over blank treatment. *ns*: non-significant.

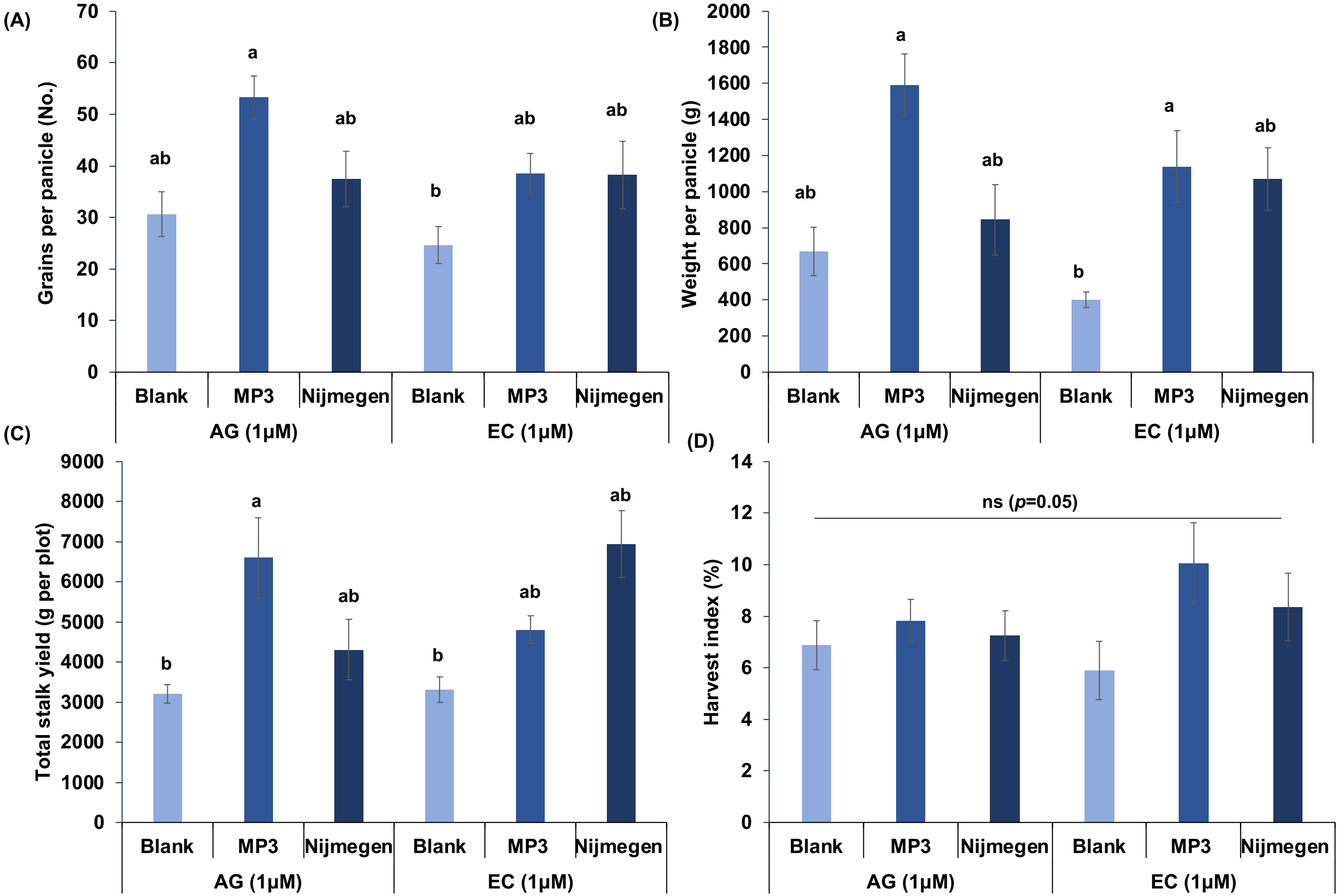

Supplementary Figure S3. Effect of various formulations of SL analogs on number of grains per panicle, panicle dry biomass, total stalk yield and harvest index from *Striga* infested Sorghum field at INERA, Burkina Faso. (**A**) Average grains per panicle, (**B**) Average weight of panicle, (**C**) Average stalk yield and (**D**) Average of harvest index from *Striga* infested Sorghum field in response to formulated MP3 and Nijmegen application. Both SL analogs of EC and AG formulations (at 1.0 µM) were applied in *Striga* infested Sorghum field. Yield and yield component were measured after 110 days of Sorghum planting. Values of each bar showed average of *Striga* emergence per plot. Data are means± SE (n=5). For each SL analog, treatments with various letters differ significantly ($p < 0.05$). Values in parenthesis are showing the percentage increase (+) or decrease (-) over blank treatment. *ns*: non-significant.
