# Supplementary material for: A New Formulation for Strigolactone Suicidal Germination Agents, towards Successful Striga Management
Source: Plants (Basel). 2022 Mar 18;11(6):808. doi: 10.3390/plants11060808 (PMC8955415; doi:10.3390/plants11060808)
Supplement: Supplementary file 1 [file plants-11-00808-s001.zip › Supplementray File S2.pdf]

Preparation Date 14-May-2020 **Revision Date** - **Revision Number 01**

1. IDENTIFICATION OF THE SUBSTANCE/PREPARATION AND OFTHE COMPANY/UNDERTAKING

Product Identifier

Product Description Nijmegen 34 EC

Recommended use of the chemical and restriction on use

Recommended use R&D material to be handled by technically qualified individuals only

Uses advised against

Details of the Supplier of the Safety Data Sheet

Supplier Address

UPL Limited
Uniphos House, Madhu Park,
Khar (W), Mumbai, India
Tel: +91-(022)- 2646-8000

E-mail address info.in@uniphos.com

Emergency Telephone Number

Company Phone Number +91-(022)- 2646-8000
Emergency telephone number +91-(022)- 2646-8000 (From 9:00 A.M. to 8:00 P.M.)

2. Hazard Identification

Classification

Flammable Liquid	Category 3 - (H226)
Skin corrosion/Irritation	Category 2 - (H315)
Eye Damage/Irritation	Category 2A - (H319)
Carcinogenicity	Category 1B – (H350)
Reproductive toxicity	Category 2 – (H361)
STOT - Single Exposure	Category 3 - (H335)
STOT - Single Exposure	Category 3 - (H336)
STOT - Repeated Exposure	Category 2 - (H373)
Aquatic Acute Hazard	Category 1 - (H400)
Aquatic Chronic Hazard	Category 2 - (H411)

Label elements

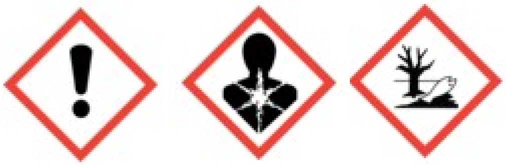

Signal word **Warning**

Hazard Statements

H226 - Flammable liquid
H315 - Causes skin irritation
H319 - Causes serious eye irritation
H335 - May cause respiratory irritation
H336 - May cause drowsiness or dizziness
H350 - May cause cancer
H361 - Suspected of damaging fertility or the unborn child
H373 - May cause damage to organs through prolonged or repeated exposure
H400 - Very toxic to aquatic life
H411 - Toxic to aquatic life with long lasting effects

Precautionary Statements

Prevention
P201 - Obtain special instructions before use
P202 - Do not handle until all safety precautions have been read and understood
P210 - Keep away from heat, hot surfaces, open flames, sparks. - No smoking
P260 - Do not breathe dust, fume, gas, mist, spray, vapors
P261 - Avoid breathing dust, fume, gas, mist, spray, vapors
P264 - Wash hands thoroughly after handling
P271 - Use only outdoors or in a well-ventilated area
P273 - Avoid release to the environment
P280 - Wear protective clothing, protective gloves, eye protection

P301 + P310 - If swallowed: Immediately call a doctor, a POISON CENTER
P302 + P352 - If on skin: Wash with plenty of water
P304 + P340 - If inhaled: Remove person to fresh air and keep comfortable for breathing
P305+P351+P338 - If in eyes: Rinse cautiously with water for several minutes. Remove contact lenses, if present and easy to do. Continue rinsing
P308 + P313 - If exposed or concerned: Get medical advice/attention
P312 - Call a doctor, a POISON CENTER if you feel unwell
P314 - Get medical advice/attention if you feel unwell
P321 - Specific treatment (see a doctor on this label)
P331 - Do NOT induce vomiting
P332 + P313 - If skin irritation occurs: Get medical advice/attention
P337 + P313 - If eye irritation persists: Get medical advice/attention
P362 + P364 - Take off contaminated clothing and wash it before reuse
P370 + P378 - In case of fire: Use alcohol resistant foam, carbon dioxide (CO2), dry extinguishing powder, Water spray to extinguish
P391 - Collect spillage

P403 + P233 - Store in a well-ventilated place. Keep container tightly closed
P403 + P235 - Store in a well-ventilated place. Keep cool
P405 - Store locked up

P501 - Dispose of contents/container to hazardous or special waste collection point, in accordance with local, regional, national and/or international regulation

Other Information
No other hazards known.

3. Composition/information on Ingredients

Chemical name	CAS-No	W/W %
Solvent naphtha	64742-94-5	40 - 60
Nijmegen	159155-03-0	1 - 5

If CAS number is "proprietary", the specific chemical identity and percentage of composition has been withheld as a trade secret.

4. First aid measures

First-aid measures

Eye contact
Hold eyes open and rinse slowly and gently with water for 15 - 20 minutes. Remove contact lenses, if present and easy to do. Continue rinsing. If eye irritation persists, get medical advice/attention.

Skin contact
Remove contaminated clothing and shoes immediately, then wash with plenty of water. Clean contaminated clothing and shoes before re-use or discard if they cannot be thoroughly cleaned. If skin irritation or rash occurs, get medical advice or attention

Ingestion
Rinse mouth. Never give anything by mouth to an unconscious person. Do not induce vomiting unless told by physician. If vomiting occurs ensure patient can breathe. If you feel unwell, get medical attention.

Inhalation
IF INHALED: Remove victim to fresh air and keep at rest in a position comfortable for breathing. Give artificial respiration if victim is not breathing. If you feel unwell, get medical attention.

Most Important Symptoms and Effects, Both Acute and Delayed

Most Important Symptoms and Effects
The most important known symptoms and effects are described in the labelling (See section 2) and/or in section 11.

Indication of immediate medical attention and special treatment needed

Notes to physician
Treat symptomatically and supportively.

5. Fire-fighting measures

Extinguishing media

Suitable extinguishing media
Water spray, dry chemical or carbon dioxide

Unsuitable extinguishing media
None known

Special hazards arising from the substance or mixture

Special Hazard
None known

Hazardous combustion products
Carbon oxides (Cox),

Protective equipment and precautions for firefighters

Wear self-contained breathing apparatus and protective suit. Use standard firefighting procedures and consider the hazards of involved material.

6. Accidental release measures

Personal precautions, protective equipment and emergency procedures

Personal Precautions
Do not eat, drink or smoke while using this product. Avoid contact with skin and eyes. Do not breathe dust/mist. Keep unnecessary people away. Ensure adequate ventilation. Isolate area. Keep out of low areas.

Environmental precautions
Consult a regulatory specialist to determine appropriate state or local reporting requirements, for assistance in waste characterization and/or hazardous waste disposal and other requirements listed in pertinent environmental permits.

Methods and material for containment and cleaning up

Methods for Clean-Up
Soak up with inert absorbent material (e.g. sand, silica gel, acid binder, universal binder, sawdust). Follow all label instruction regarding spill clean-up and residual disposal methods.

Reference to other sections
Refer to Section 8 - Exposure Controls/Personal Protection and Section 13 - Disposal Considerations.

7. Handling and Storage

Precautions for safe handling

Handling
Avoid contact with skin and eyes. Do not breathe dust/mist. Ensure adequate ventilation. Wash thoroughly after handling. Avoid release to environment. Empty containers may contain hazardous residues. Do not reuse empty container.

Conditions for safe storage, including any incompatibilities

Storage
Store in accordance with local regulation. Store in original container. Keep containers tightly closed in a cool, well-ventilated place. Keep away from incompatible material. Do not store in unlabelled containers. Do not store material near food, feed or drinking water.

Incompatible materials
Strong oxidizing agent

8. Exposure Controls/Personal Protection

Exposure Guideline
Apply technical measures to comply with the occupational exposure limit.

Engineering controls
Ensure adequate ventilation, especially in confined areas. If applicable, use process enclosures, local exhaust ventilation, or other engineering controls to maintain airborne levels below recommended exposure limits. If exposure limits have not been established, maintain airborne levels to an acceptable level.

Personal protective equipment

Eye/Face Protection
Avoid contact with eyes. Where there is potential for eye contact have eye flushing equipment available. Tightly fitting safety goggles. Face-shield.

Skin/Hand protection
Wear protective gloves/clothing. Long-sleeved clothing

Respiratory protection
When workers are facing concentrations above the exposure limit they must use appropriate certified respirators.

Environmental exposure controls
Prevent further leakage or spillage if safe to do so. Do not let product enter drains. Discharge into the environment must be avoided. Local authorities should be advised if significant spillage cannot be contained.

General hygiene considerations
Do not eat, drink or smoke when using this product. Keep away from food, drink and animal feeding stuffs. Wash hands before breaks and immediately after handling the product. Remove and wash contaminated clothing before re-use.

9. Physical and Chemical Properties

Information on basic physical and chemical properties

**Appearance**	homogeneous free slowing liquid
**Physical state**	Liquid
**Odor**	No information available
**Color**	Pale yellow to light brown
**Odor threshold**	No information available.

Property	VALUES	Remarks/ Method
**pH**	4-5	
**Melting point/freezing point**	No information available	
**Boiling Point/Range**	No information available	
**Flash Point**	>48 °C	
**Flammability (solid, gas)**	No information available	
**Upper/lower flammability or**	No information available	
**Explosive limit**	No information available	
**Density**	0.96 – 1.05 at 20°C	
**Vapor density**	No information available	
**Vapor pressure**	No information available	
**Water solubility**	No information available	
**Solubility in Other Solvents**	Soluble in Xylene, Naphtha, kerosene	
**Partition coefficient: n-octanol/water**	No information available	
**Autoignition temperature**	No information available	
**Decomposition temperature**	No information available	
**VOC Content**	No information available	
**Kinematic Viscosity**	No information available	
**Oxidizing properties**	No information available	
**Explosive properties**	No information available	
**Surface Tension**	No information available	

10. Stability and Reactivity

Reactivity

No information available

Chemical stability

Stable under recommended storage conditions.

Possibility of hazardous reaction

None under normal processing.

Conditions to avoid

Avoid heat, flames and spark. Avoid contact with incompatible material.

Incompatible Materials

Strong oxidizing agents

Hazardous decomposition products

Thermal decomposition can lead to release of irritating or toxic fumes (or gases) in fire.

Thermal hazardous decomposition products: Carbon oxides (Cox), Tin oxide

11. Toxicological Information

Information on Toxicological Effects

LD50 Oral = Not Data Available
LD50 Dermal = Not Data Available
LC50 Inhalation = Not Data Available

Local effect

**Inhalation**	No information available
**Eye contact**	Causes serious eye irritation. (Based on major component)
**Skin contact**	Causes Skin irritation. (Based on major component)
**Ingestion**	Based on available data, the classification criteria are not met.

Chronic toxicity

**Skin Corrosion/Irritation**	No information available
**Eye damage/irritation**	No information available
**Sensitization**	No information available
**Mutagenic effects**	No information available
**Carcinogenic effects**	No information available
**Reproductive effects**	No information available
**STOT - Single Exposure**	May cause drowsiness or dizziness.
**STOT - repeated exposure**	May cause damage to organs through prolonged or repeated exposure.
**Aspiration hazard**	No information available

12. Ecological Information

Ecotoxicity

- Very toxic to aquatic life
- Toxic to aquatic life with long lasting effects (Based on major component)

LC50/Fish/96hr = No information available

EC50/Daphnia/48hr = No information available

EC50/Alga/72hr = No information available

Persistence and Degradability

No information available.

Bioaccumulative Potential

No information available.

Other Adverse Effects

No information available.

13. Disposal Considerations

Waste Treatment Methods

**Waste Disposal Method**	Dispose of in accordance with applicable Federal, State, local laws and regulations.
**Contaminated packaging**	Empty container should be taken for local recycling, recovery or waste disposal. Do not re-use container. Avoid dispersal of spilt material and runoff and contact with soil, waterways, drains and sewers.

14. Transport Information

IMDG/IMO

**14.1 UN-No**	UN 1993
**14.2 Proper Shipping name**	FLAMMABLE LIQUID, N.O.S. (petroleum naphtha)
**114.3 Hazard class**	3
**14.4 Packing group**	III
**14.6 Environmental Hazard**	Marine pollutant

IATA/ICAO

**14.1 UN-No**	UN 1993
**14.2 Proper Shipping name**	FLAMMABLE LIQUID, N.O.S. (petroleum naphtha)
**114.3 Hazard class**	3
**14.4 Packing group**	III
**14.5 Environmental Hazard**	Marine pollutant

15. Regulatory Information

International Inventories

**USINV**	-
**TSCA**	-
**EINECS/ELINCS**	
**DSL/NDSL**	-
**PICCS**	-
**ENCS**	-
**China**	-
**AICS**	-
**KECL**	-

- : Not listed/Determines

Legend

USINV - Total TSCA/FIFRA Inventory
TSCA - United States Toxic Substances Control Act Section 8(b) Inventory
DSL/NDSL - Canadian Domestic Substances List/Non-Domestic Substances List
EINECS/ELINCS - European Inventory of Existing Chemical Substances/European List of Notified Chemical Substances
PICCS - Philippines Inventory of Chemicals and Chemical Substances
ENCS - Japan Existing and New Chemical Substances
AICS - Australian Inventory of Chemical Substances
KECL - Korean Existing and Evaluated Chemical Substances

16. Other Information

**Preparation Date**	14-MAy-2020
**Revision date**	

Preparation Date 14-May-2020 **Revision Date** - **Revision Number 01**

Revision Summary

Disclaimer
UPL, Limited. believes that the information and recommendations contained herein (including data and statements) are accurate as of the date hereof. NO WARRANTY OF FITNESS FOR ANY PARTICULAR PURPOSE, WARRANTY OF MERCHANTABILITY OR ANY OTHER WARRANTY, EXPRESSED OR IMPLIED, IS MADE CONCERNING THE INFORMATION PROVIDED HEREIN. The information provided herein relates only to the specific product designated and may not be valid where such product is used in combination with other materials or in any process. Further, since the conditions and methods of use are beyond the control of UPL, Limited. and UPL Limited. expressly disclaims any and all liability as to any results obtained or arising from any use of the product or reliance on such information.

End of Safety Data Sheet
